# Bispectrum and Recurrent Neural Networks: Improved Classification of Interictal and Preictal States

**DOI:** 10.1038/s41598-019-52152-2

**Published:** 2019-10-30

**Authors:** Laura Gagliano, Elie Bou Assi, Dang K. Nguyen, Mohamad Sawan

**Affiliations:** 1 Polystim Neurotech Lab, Institute of Biomedical Engineering, Polytechnique Montreal, Montreal, QC Canada; 2 University of Montreal Hospital Center (CHUM), University of Montreal, Montreal, QC Canada; 3 CenBRAIN, School of Engineering, Westlake University, Hangzhou, 310024 China; 4 Institute of Advanced Study, Westlake Institute for Advanced Study, Hangzhou, 310024 China

**Keywords:** Biomedical engineering, Epilepsy

## Abstract

This work proposes a novel approach for the classification of interictal and preictal brain states based on bispectrum analysis and recurrent Long Short-Term Memory (LSTM) neural networks. Two features were first extracted from bilateral intracranial electroencephalography (iEEG) recordings of dogs with naturally occurring focal epilepsy. Single-layer LSTM networks were trained to classify 5-min long feature vectors as preictal or interictal. Classification performances were compared to previous work involving multilayer perceptron networks and higher-order spectral (HOS) features on the same dataset. The proposed LSTM network proved superior to the multilayer perceptron network and achieved an average classification accuracy of 86.29% on held-out data. Results imply the possibility of forecasting epileptic seizures using recurrent neural networks, with minimal feature extraction.

## Introduction

Epilepsy is one of the most prevalent neurological conditions in the world affecting about 69 million people of all ages (World Health Organization). While causes of epilepsy can vary (genetic mutations, brain malformations, tumors, trauma, stroke etc.), the end result is the occurrence of recurrent disabling seizures. The first line of treatment to control seizures consists of chronic antiepileptic drug therapy; unfortunately, more than 30% of patients are pharmacoresistant. Although epilepsy surgery is a viable alternative treatment option, recourse to resective brain surgery is still relatively low due to accessibility, fear of complications, and variable success rates (subject to the complexity of the case)^1,2^. One of the most limiting and detrimental aspects of epilepsy is the unpredictable nature of seizures which significantly affects the quality of life of patients by instilling a constant sense of worriment and anxiety as well as limiting their autonomy^3^. Over the past three decades, researchers have investigated the possibility of anticipating seizures^4–6^ as an alternate epilepsy treatment strategy for refractory patients. In contrast to seizure detection, seizure prediction aims to detect the period preceding the seizure onset, called the preictal state, before any clinical manifestations. The ability to accurately forecast seizures gives promise for the development of closed-loop advisory/intervention systems. Such devices would continuously record brain electrical activity and, upon prediction of an upcoming seizure, warn the patient/caretaker/medical personnel to take appropriate measures or even automatically trigger seizure abortion techniques such as an inhibitory electrical impulse^3^.

The ability to accurately identify the preictal state (period preceding seizure onset) relies on the adequate identification of intracranial electroencephalography (iEEG) features capable of tracking brain dynamics during the transition to seizures. Notwithstanding that several endeavors have been made in an attempt to find a unique precursor of epileptic seizure activity, notably spectral band power, no single feature has been capable of universally capturing brain dynamics prior to seizure onset^7^. With traditional techniques, several types of features must be extracted and combined in a classifying algorithm to preserve the spectral, temporal and spatial preictal signatures of seizure mechanisms^5,8,9^. While such algorithms are successful in predicting seizures before their onset, they require extracting a relatively high number of distinct features to adequately capture different preictal iEEG signatures. This would in turn require the development of multiple different extraction blocks in the case of a hardware implementation. In a recent review of seizure prediction, Freestone *et al*. highlight the need for more generalized and less computationally complex forecasting algorithms in order to be translated to practical clinical applications^10^.

Recently, more complex iEEG-extracted features measuring cross-frequency coupling (CFC) have been shown to successfully characterize neuronal activity and brain dynamics^11,12^. In a follow-up study, we evaluated the amplitude modulation of high frequency (gamma band) oscillations by low-frequency (delta and theta band) phase and showed the existence of a statistically significant preictal period characterized by bilateral changes in this type of CFC called phase-amplitude coupling (PAC)^13^. The advantage of using cross-frequency coupling measures rather than spectral-band power alone is that it provides information on interactions between the frequency bands of interest and hence captures the epileptic brain network activity. Our group has later extended our search for CFC precursors by exploring the feasibility of using the bispectrum, a measure of nonlinear multi-frequency interactions, for seizure prediction^14^. It was initially selected for evaluation as a novel precursor because it quantifies nonlinear cross-frequency interactions which encompass both phase and amplitude of frequency components. Interestingly, quantitative bispectrum-extracted features exhibited significant changes prior to seizure onset and, when used as inputs to a feedforward artificial neural network classifier, demonstrated promising seizure prediction results in canine epilepsy^14^.

While the use of artificial intelligence with iEEG for the prediction of epileptic seizures dates back to the 1970s, the continuously growing field of machine learning has more recently tremendously benefitted seizure prediction thanks to modern computation and data storing technologies^5,6^. Artificial neural networks are a form of machine learning in which simple mathematical operations are combined with non-linear activations to model complex representations of input data and learn relations between these representations and a certain output^15^. In the case of seizure prediction, artificial neural networks are trained to represent raw iEEG or iEEG-extracted features from short segments of interictal (non-seizure activity) and preictal recordings and learn to map these representations to their class label (preictal or interictal)^6^. This type of approach allows for prediction algorithms to have a general pipeline while they are trained in a subject-specific manner (meaning that they learn personal preictal patterns), an important attribute for accurate prediction^10^. More specifically, in line with recent studies and literature on seizure prediction, a neural network classifier was trained and tested on data from a single animal to account for the high inter-subject variability (e.g. different seizure types, onset patterns, etc.)^4–8,10,16^. This strategy was supported and encouraged by the Kaggle Melbourne seizure prediction competition (www.kaggle.com/c/melbourne-university-seizure-prediction) and the epilepsy ecosystem^17^ which accepts and recommends the design of subject-specific algorithms to optimize performance for each animal. In^14^, we proposed a simple multilayer perceptron network which classifies bispectrum features extracted from long-term canine recordings. Preliminary findings regarding the feasibility of distinguishing between and automatically classifying preictal and interictal segments of iEEG based solely on bispectrum-extracted features showed promising performances albeit temporal dynamics of iEEG signals were not considered.

In this work, we extend these investigations to the use of a classification scheme able to learn temporal dynamic behavior of preictal time sequences. Thus, a recurrent neural network was used rather than the traditional feedforward neural network^18^. A recurrent neural network receives information from previous samples through feedback connections to classify the current input, making it ideal for classification of time-sequences such as audio and video recordings^15,19^. Recent studies have shown the appropriateness of long short-term memory (LSTM) recurrent neural networks for EEG signal classification, including seizure detection and prediction^20–22^. While these studies present optimistic EEG classification results, the LSTM architecture is preceded by various hand-engineered feature extraction processes or an extensive convolutional neural network for automatic feature extraction. Our proposed pipeline requires only the bispectral analysis from raw iEEG recordings and a single-layer LSTM network for classification of 5-min recordings. The remaining sections present the bispectral feature extraction and LSTM architecture. Training strategies and comparison of statistical classification performances to previous work are then discussed. We conclude with an interpretation of the current findings and proposal of prospective studies.

## Materials and Method

**Figure 1:**
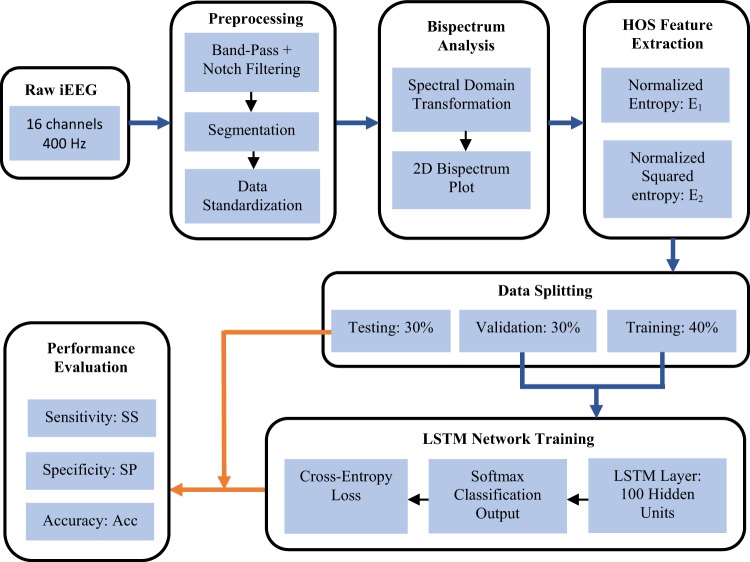
Schematic block diagram of proposed signal processing, neural network training, and evaluation methodology.

The methodology followed in this work can be divided into the data processing and feature extraction followed by classification algorithm training and performance evaluation. These steps are elaborated in this section and presented schematically in Fig. 1.

### Bispectral feature extraction

#### Database

Higher-order spectral features were extracted from long-term, bilateral canine iEEG recordings. Raw signals were freely accessed online via the NIH-sponsored international electrophysiology portal (https://www.ieeg.org/). Similar to recent seizure forecasting investigations based on canine iEEG, this database was chosen for this study because canine epilepsy has been demonstrated as a suitable model for human epilepsy^9,14,23,24^ and because it provides longer (up to 1 year per dog) recordings than human databases which generally include short-term recordings (∼2 weeks) from patients admitted for epilepsy surgery evaluation. The NeuroVista ambulatory monitoring device was used to acquire continuous iEEG data in 3 dogs with naturally occurring focal epilepsy^25^. Dogs were implanted with bilateral contacts following a standardized protocol^24^ and data acquisition studies were approved by the University of Minnesota Institutional Animal Care and Use Committee where the animals were kept. All procedures were performed in accordance with relevant regulations and guidelines. Recordings, acquired at 400 Hz, were continuous in nature and spanned several months; however, segmentation was performed prior to feature extraction. Since there exists no gold standard defining the preictal period length, 1-hour long preictal periods (with 5-minute intervention time) were used, in line with previous studies and guidelines proposed by the American Epilepsy Society seizure prediction challenge^5^ and the epilepsy ecosystem^17^. For each dog, only seizures which were preceded by 65 minutes of uninterrupted preictal recordings over the 16 channels were selected and used. This resulted in 17 preictal hours for dogs 1 and 2 and 11 preictal hours for dog 3 for a total of 45 preictal hours (coming from 45 seizures). To prevent overfitting during the training of the neural networks, data from preictal and interictal classes were balanced (resulting in the same number of segments in each class). Furthermore, for each 1-hour preictal segment, a 1-hour interictal segment was randomly chosen from the entire interictal data at least 4 hours before or after a seizure.

#### Bispectrum analysis

A bispectrum analysis is a 2-dimensional mapping of nonlinear interactions between the various frequency components of a time series. As denoted in Eq. (1), this measure of quadratic coupling is computed for a pair of oscillatory components, *f*_1_ and *f*_2_, and is based on the Fourier Transform of higher-order correlation functions which consider the pair *f*_1_, *f*_2_, and their harmonic component *f*_3_ = *f*_1_ + *f*_2_.

1 $$Bis({f}_{1},{f}_{2})={\mathrm{lim}}_{T\to \infty }(\frac{1}{T})E[X({f}_{1}+{f}_{2}){X}^{\ast }({f}_{1}){X}^{\ast }({f}_{2})]$$

where X(f) denotes the value of the Fourier transform of x(t) at frequency f, and E denotes the arithmetic average estimator.

Consequently, the value plotted at each intersection (*f*_1_, *f*_2_) quantifies the degree of higher-order oscillatory correlation between *f*_1_, *f*_2_, and *f*_3_. This measure simultaneously conceals information on both phase and magnitude coupling between components which eliminates the need for multiple distinct feature extraction techniques. Bispectrum computation was performed on 30-sec non-overlapping windows of preprocessed iEEG recordings.

Preprocessing consisted of band-pass (0.5–180 Hz) and notch (60 Hz) filtering followed by data standardization (i.e. rescaling data to have a mean equal to 0 and standard deviation of 1). Preprocessing and bispectrum analysis were parallelized offline using both the Higher-Order Spectral Analysis (HOSA) Matlab toolbox and Matlab’s parallel computing toolbox on a 16-core (1024 GB RAM, 2.80 GHz) computer.

#### Higher-order spectral features

**Figure 2:**
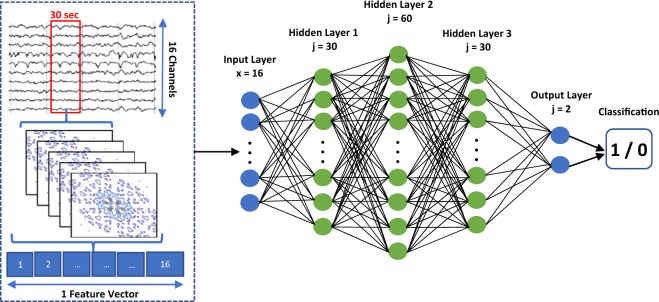
Multilayer perceptron classifier proposed in Bou Assi *et al*., Scientific Reports 2018^14^.

According to recent articles on the practical challenges in translating seizure prediction algorithms into clinical intervention/advisory systems, computational complexity was considered as a major obstacle^8,10^. The bispectrum analysis is not as computationally expensive as other previously explored measures of cross-frequency coupling^12^. However, given the analyzed frequency range of interest, each 30-sec window yields a 2D array of size 4096 × 4096. The bispectrum density plots obtained for each channel are illustrated in the feature extraction block of Fig. 2. Designing a neural network to classify such a large input variable would be both impractical and unnecessary since the quantity of operations would not be implementable on chip. To optimize both the size and computational expense of our proposed neural network classifier, quantitative features were extracted from the 2D bispectrum density plots and fed as input to the neural network classifier.

In^14^, we performed an extensive statistical analysis on three bispectral features to evaluate the significance of the preictal changes in these features. The two features which have displayed most significant differences were combined to classify interictal and preictal iEEG segments in this study. These quantitative features are the normalized entropy (2), noted E1 and the normalized squared entropy (4), noted E2. Features are calculated as follows, where N represents the total number of data points in the non-redundant region of interest Ω of the bispectrum plot.

2 $${E}_{1}=-\,{\sum }_{L}({p}_{i}\,\log \,{p}_{i}),\,\,i=1,2,\,\ldots ,\,L$$

3 $${p}_{i}=\frac{|B({f}_{m},{f}_{n})|}{{\sum }_{\varOmega }|B({f}_{m},{f}_{n})|}\,\,\forall \,{f}_{m},{f}_{n}\in {\Omega }$$

4 $${E}_{2}=-\,{\sum }_{L}({q}_{i}\,\log \,{q}_{i}),\,\,i=1,2,\,\ldots ,\,L$$

5 $${q}_{i}=\frac{{|B({f}_{m},{f}_{n})|}^{2}}{{\sum }_{\Omega }{|B({f}_{m},{f}_{n})|}^{2}}\,\,\forall \,{f}_{m},{f}_{n}\in \Omega $$

where i = 0, 1, …, L − 1; L is the total number of possible pairs in the non-redundant region of the bispectral density array Ω.

**Figure 3:**
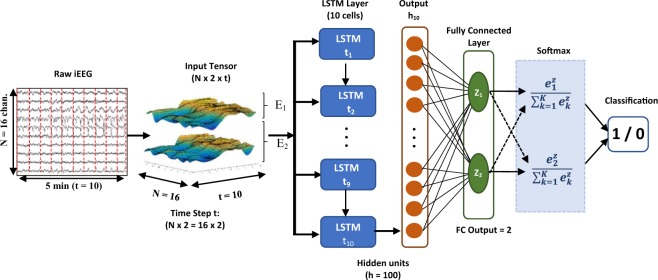
LSTM neural network architecture. Input layer consists of 10 sequential time steps (10 × 30 sec = 5 min), each containing 32 feature-channel combinations (2 features × 16 channels). LSTM layer consists of 10 LSTM cells each having 100 hidden units. FC = fully connected layer taking output matrix (h_10_) from 10^th^ LSTM cell. Output layer consists of 2 nodes for binary classification.

The aforementioned features were extracted from all available iEEG channels (n = 16) yielding a total of 32 electrode-feature combinations for each 30-sec window. The HOS feature extraction can be seen in Fig. 3 which illustrates an example of the 32 features for 10 sequential time steps (5 min) fed as input to the recurrent neural network classifier. Figure 1 displays the block diagram of the implemented analytical framework from signal acquisition to automatic classification and performance evaluations.

### Seizure prediction algorithm

#### Multilayer perceptron architecture

In our previous work^14^, our group assessed the feasibility of automatically identifying the preictal period, from the same canine database, using only one bispectrum feature as input to a simple multilayer perceptron network. The proposed architecture, shown in Fig. 2, consists of a 16-node input layer (1 feature for each channel) followed by three hidden layers of 30, 60 and 30 nodes and a 2-node output layer which allows for a binary classification decision. Rectified linear unit (ReLU) activation function was used for all nodes. In this study, one neural network per feature was designed for each dog and trained using stochastic gradient descent and early stopping on training and validation sets consisting of the first 40% and 30% of the dog’s seizures. This allowed for a comparison of different bispectral features’ abilities to classify single 30-sec iEEG samples in a subject-specific manner.

#### Recurrent neural network architecture

In this work, the bispectrum features were implemented into a seizure prediction framework. To evaluate the classification performance of an artificial neural network using only 2 bispectral features as input, a recurrent neural network was chosen for its ability to treat sequential input data. Long short-term memory neural networks are a type of recurrent neural network which use hidden memory cells to retain long-term dependencies in sequential data, such as time sequences, making them more appropriate for classification of EEG recordings^18^.

In line with our objective of minimizing internal variables and the computational complexity of the classifier, a simple single-layer LSTM architecture, illustrated in Fig. 3, was implemented for classification of 5-minute long interictal and preictal iEEG sequences. The input layer consists of 10 sequential feature vectors, each containing the 32 bispectral features (2 features from each channel) from one 30-second extraction window for a total of 5 minutes.

The single unidirectional LSTM layer consists of 10 sequential standard LSTM cells. Each cell takes in the input vector from a single time step as well as the cell state and hidden state matrix from the previous cell and executes a series of operations which make up the forget, update and output gates of the cell. These gates learn both patterns and long-term dependencies by learning to retain, forget and update certain information of the cell state before passing it to the next cell. The mathematical equations involved in the LSTM cells are detailed in^15^. A hyperparameter optimization was done by varying the number of sequential time-steps and the size of the hidden memory cell (hidden state). Ten time steps with 100 hidden memory cells each (h = 100) was found to yield optimal validation results across the 3 subjects. The 10^th^ and final LSTM cell output is followed by one fully connected layer with an output of 2 and one final output layer which uses the Softmax activation function and leads to the binary classification (i.e. interictal or preictal) of the 5-min sequence. Softmax was favored for the output activations since it returns a probability for each class which can later be used in a regularization post-processing.

#### LSTM training and data splitting

**Figure 4:**
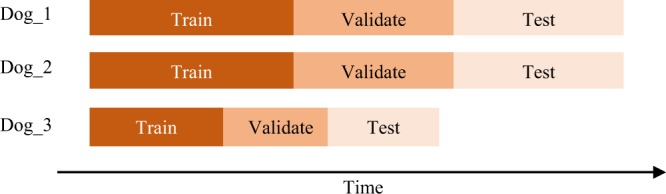
Data splitting technique applied to prevent temporal contamination of validation and testing data. Data was split temporally on a seizure-per-seizure basis where training validation and testing corresponds to 40%, 30%, and 30% respectively.

LSTM networks were trained, validated and tested in a subject-specific manner where data was temporally split into 40% training set, 30% validation set, and 30% testing set for a total of 45 seizures. To avoid biased classification results, training, validation and testing datasets were temporally split on a seizure-per-seizure basis^9,14,26^. In other words, all 5-minute segments from any given preictal hour were used either for training, validation or testing. This way, the classifier is trained and validated only on the first 70% of seizures and tested on the last 30% of seizures as illustrated in Fig. 4. This form of held-out testing avoids overfitting due to time correlations between train and test samples and is more representative of the clinical application of the algorithm which would predict future unseen seizures. While other cross-validation approaches can be used (eg. N-fold cross-validation), in this work, a held-out validation strategy was favored since it imitates a real clinical scenario: train and validate on early acquired recordings and test on future unseen data. Various training parameter combinations were tested, and the optimal parameter configuration was used for all three dogs. The three classifiers were trained using cross-entropy loss function on a minibatch of 24 sequences, for a maximum of 4800 iterations. Backpropagation was optimized using the Adaptive Momentum Estimator (ADAM) with an initial learning rate of 0.001. To prevent divergence during backpropagation, a variable learning rate was applied^15^. The learning rate dropped by a factor of 0.1 every 50 epochs.

Since training and validation loss converged after 2000 iterations for all three dogs and the ADAM optimizer was used, no early stopping was required.

#### Classification performance evaluation

A quantitative evaluation of the classification performances of the three trained LSTM neural networks was done using three statistical metrics: sensitivity, specificity, and classification accuracy. Here, standard metrics were used on classification output on final training samples and on the held-out test set. Sensitivity (SS) is defined as the ratio of correctly classified positive (preictal) samples (i.e. true positives = TP) to the total number of positive samples (P) (6). Specificity (SP) can be described as the true negative rate which is the proportion of the negative samples (N) which are correctly labeled as such (TN) (7). Finally, the classification accuracy (Acc.) is a combination of both sensitivity and specificity (8).

6 $$SS=\frac{TP}{P}\times 100 \% $$

7 $$SP=\frac{TN}{N}\times 100 \% $$

8 $$Acc.=\frac{TP+TN}{P+N}\times 100 \% $$

## Results and Discussion

### Subject-Specific EEG classification results

**Figure 5:**
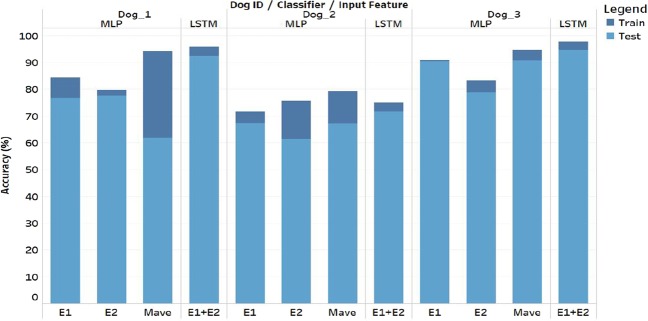
Train and test classification accuracies for all MLP^14^ and LSTM networks. Horizontal axis represents input features. Mave: Average magnitude; E1: Normalized entropy; E2: Normalized squared entropy.

In a proof-of-principle study, our group has previously demonstrated that there exists a statistically significant change in bispectrum measures prior to seizure onset and that a simple multilayer perceptron (MLP) neural network classifier is capable of learning to distinguish between preictal and interictal 30-second iEEG recordings based on single iEEG bispectral features from 16 channels^14^. As a follow-up, this study attempted to improve the classification performance of neural networks based on bispectral features using the same canine database with a different artificial neural network architecture. In contrast to our previous work using an MLP, the LSTM classifier was fed two bispectral features rather than one and was trained to classify 10 sequential 30-second iEEG segments which allows for temporal patterns in the features to be recognized. Train and test accuracies from both studies are presented in Table 1and Fig. 5. In^14^, three neural networks were tested for each dog (one for each bispectral feature). For comparison purposes, the results reported in the table are those from the best performing feature for each dog while Fig. 5 contains both train and test accuracies for all 3 MLP networks. Table 1 shows that our newly proposed recurrent neural network framework outperformed the MLP classifier in terms of test classification accuracy for all three dogs with respective average test accuracies of 86.2% and 78.11%. The improved classification accuracy obtained with a generalized learning algorithm (i.e. exact same features used across all subjects) over a more tailored subject-specific feature selection suggests that the additional information contained in the temporal domain captures a more generalized preictal activity better than a single time step. This can also be visualized in Fig. 5 where the test accuracy for the single LSTM framework is greater than the best performing MLP for each dog. The variability in the classification accuracies between the three dogs, in both^14^ and this study, highlights the importance of patient-specific learning in the seizure prediction problem^10^. Moreover, Fig. 5 also demonstrates that for all dogs, the difference between the train and test accuracies is consistently small for the LSTM networks compared to the variable MLPs. The difference between the train and test accuracies is a measure of the level of overfitting. A small difference between the two indicates that the network is learning general patterns in the signals rather than memorizing the training data and hence the LSTM framework is consistently more generalizable to unseen data.

**Table 1:** Binary iEEG classification results of LSTM compared to previously used MLP^14^.

Subject ID	# Sz.	This Work LSTM				Bou Assi *et al*.^14^ MLP	
		*Test SS (%)*	*Test SP (%)*	*Accuracy (%)*		*Accuracy (%)*	
				*Train*	*Test*	*Train* ^a^	*Test* ^a^
1	17	86.67	98.33	95.83	92.50	84.23	76.71
2	17	56.29	87.05	75.00	71.67	71.71	67.23
3	11	91.67	97.92	95.69	94.71	90.89	90.40
**Total**	**45**	**78.21**	**94.43**	**88.84**	**86.29**	**82.28**	**78.11**

^a^For each dog, three neural networks were trained (one per feature), the results shown correspond to the feature yielding the highest classification accuracy.

Interestingly, for all three dogs, specificity values are greater than sensitivity values with average test sensitivity and specificity of 78.21% and 94.43% respectively. Once again, the difference between the sensitivity and specificity varies between dogs. This may be caused by mislabeled preictal data. High specificity rates indicate that the LSTM is capable of identifying interictal segments with ease. However, a portion of the segments labeled as preictal may be mistakenly classified as interictal. These results suggest that the accepted hour-long preictal period may not correspond to the true physiological preictal period for all subjects. For example, dog 3 achieved a sensitivity of 91.67% while dog 2 achieved a sensitivity of only 56.26% meaning that dog 2 may manifest a shorter physiological preictal period and that some of the preictal data segments are mislabeled, meaning they resemble interictal segments more than they do preictal segments. It would therefore be of great interest to statistically optimize the preictal duration in a subject-specific manner in future studies attempting to classify interictal and preictal iEEG. Although clinical metrics are of great importance to the evaluation of the clinical relevance of seizure prediction algorithms, implementing post-processing or regularization to the classification results goes beyond the scope of this manuscript.

Furthermore, while other studies exploiting LSTM networks for epileptic EEG classification tasks present optimistic results, the algorithms are preceded by either extensive hand-engineered feature extraction^21^, a very large raw iEEG input tensor^22^ or an additional convolutional neural network for feature extraction^20^. In the latter study^20^, the authors used deep learning approaches to train a classifier able to identify seizure epochs. While this is possible in seizure detection studies which employ generalized algorithms, the use of subject-specific strategies (seizure prediction) limit the amount of available training data. On the other hand, Tsiouris *et al*.^21^ proposed an LSTM-based classifier to distinguish between preictal and interictal segments and reported promising classification results compared to those achieved by convolutional neural network (CNN)-based algorithms.

Their conclusions concord well with our findings regarding the improved epileptic iEEG classification accuracies obtained with recurrent algorithms, which recognize time patterns in the signal. However, scalp EEG was used and shorter 15 and 30-minute preictal periods were adopted which restricts direct comparison to this work’s results. In a recent study using a single LSTM layer for epileptic vs non-epileptic EEG classification, Hussein *et al*.^22^ reported optimistic classification sensitivity (100%) highlighting the appropriateness of using temporal patterns for EEG classification tasks. While their proposed algorithm does not require any feature extraction, their goal was to identify epileptic EEG from healthy EEG which cannot be translated to seizure prediction due to the difficulty in identifying the preictal period. We introduce in this work a classification algorithm which takes advantage of the long-term memory and recurrent nature of LSTM networks and the physiological preictal signature of bispectral measures to provide a general yet highly subject-specific preictal classification with minimal feature extraction. More specifically, only 2 bispectrum-extracted features per channel (2 × 10 time steps) were fed as input to a single layer LSTM for subject-specific preictal vs interictal classification. As suggested in recent reviews on seizure prediction^6,8,19^, technological advances in the field of machine learning, which allow for more advanced classifiers, were investigated and shown to meet the need for more generalized frameworks (same minimal feature extraction and classifier architecture for each dog). Using a generalizable feature such as the bispectrum, despite its complexity, removes the need for subject-specific feature selection and extraction rendering the classification framework more practical for hardware design and implementation. To further optimize the computational expense of the algorithms, future prospective studies include the proper selection of channels either by statistical analysis or other measures such as functional connectivity as in^9^. The used canine database includes bilateral recordings from only 16 electrode contacts however, human recordings are often acquired in local regions using high-density electrodes which would require channel selection for dimensionality reduction.

Furthermore, while bispectral analysis shows promise as a seizure precursor capable of characterizing the preictal state, recent studies investigating other iEEG-based biomarkers of seizure initiation have also opened valuable avenues for future studies. Examples include the use of high frequency EEG activity for the localization of the seizure onset zone (SOZ)^27^ and the study of epileptic brain networks for both SOZ localization^28,29^ and seizure onset detection^30^ which go hand-in-hand. As recent technological advancements allow for faster computing and big data storage, more in depth analyses have highlighted the importance of using multivariate measures such as functional and effective connectivity to understand and track epileptic network dynamics during and before seizure^29,31^. Greater understanding of epileptic network dynamics could translate to improved SOZ localization as well as seizure detection and prediction and should hence be further explored in future studies.

Adapting this iEEG classification algorithm to continuous iEEG recordings and implementing it in a real-time seizure forecasting pipeline, as proposed by Brinkmann *et al*.^24^, would be of great value to the field of seizure prediction.

Results showed promising performances in classifying preictal and interictal states from canine iEEG recordings. While canine epilepsy has been established as a good model for human epilepsy, care should be taken before generalizing findings. More specifically, results should be reproduced in recordings from human patients with epilepsy.

## Conclusion

Two main limitations currently faced by the seizure prediction research community are the lack of generalized algorithms and the difficulty in translating computationally complex forecasting frameworks to real-time closed-loop systems. In leveraging artificial recurrent neural networks and higher-order spectral features, this work offers a novel approach to these two limitations. Statistical evaluation of the LSTM classifier performance shows that combining nonlinear CFC with the temporal patterns is both appropriate and promising for subject-specific seizure prediction. Prospective studies evaluating the clinical significance as well as the feasibility of integrating the proposed algorithm into an implantable device would be a milestone in the development of an implantable closed-loop seizure forecasting system.
